# Resolution of severe autoimmune hemolytic anemia and thrombocytopenia associated with *Plasmodium vivax* malaria without corticosteroid therapy: A case report

**DOI:** 10.1097/MD.0000000000049968

**Published:** 2026-07-31

**Authors:** Abdu Aldarhami, Rahmah Alzaylaee, Ibrahim Awadh, Raja Sassi, Hassan Alzahrani, Ali Alsamiri, Kamal Ismail, Nizar H. Saeedi, Mohammed M. Jalal, Abdullah Yahya Alrashdi, Abdulrahman S. Bazaid

**Affiliations:** a Department of Microbiology and Parasitology, Faculty of Medicine, Umm Al-Qura University, Al-Qunfudah, Saudi Arabia; b Laboratory and Blood Bank Department, Qunfudah General Hospital, Qunfudah, Saudi Arabia; c Department of Medical Laboratory Technology, Faculty of Applied Medical Sciences, University of Tabuk, Tabuk, Saudi Arabia; d Department of Internal Medicine, Qunfudah General Hospital, Qunfudah, Saudi Arabia; e Department of Medical Laboratory Science, College of Applied Medical Sciences, University of Hail, Hail, Saudi Arabia.

**Keywords:** autoimmune hemolytic anemia, case report, malaria, *Plasmodium vivax*, thrombocytopenia

## Abstract

**Rationale:**

Autoimmune hemolytic anemia is a rare but potentially life-threatening complication of *Plasmodium vivax* malaria. Most reported cases required corticosteroid therapy in addition to antimalarial treatment.

**Patient concerns:**

We report the case of a 28-year-old Ethiopian man who presented with fever, pallor, and fatigue.

**Diagnosis:**

Laboratory evaluation revealed severe anemia (hemoglobin 6 g/dL), thrombocytopenia (88,000/µL), hyperbilirubinemia, and a positive direct antiglobulin test. Peripheral smear confirmed *P vivax* infection with 2% parasitemia. Comprehensive immunohematological testing, including direct antiglobulin test, elution, adsorption, and extended antigen typing, confirmed immune-mediated hemolysis and excluded alloantibody-related incompatibility.

**Interventions:**

The patient received 3 compatible blood transfusions and was treated with intravenous artesunate followed by primaquine, without corticosteroids or platelet transfusion.

**Outcomes:**

By day 5, hemoglobin improved to 10.2 g/dL, platelets normalized, and malaria smears were negative. He was discharged in stable condition.

**Lessons:**

This report highlights an unusual presentation of *P vivax* malaria complicated by severe autoimmune hemolytic anemia and thrombocytopenia, which resolved with antimalarial therapy alone, without immunosuppressive therapy, including corticosteroids. This finding suggests that corticosteroids may not always be necessary in certain cases and underscores the importance of early recognition and comprehensive immunohematological evaluation in malaria-related cytopenias.

## 1. Introduction

Malaria is a major global health problem and remains one of the most prevalent parasitic infections worldwide. Hematological complications are common in malaria and include anemia and thrombocytopenia, particularly in *Plasmodium falciparum* and *Plasmodium vivax* infections.^[1]^ Recent reviews have summarized that immune-mediated destruction, complement activation, and autoantibody formation contribute significantly to these hematological complications across *Plasmodium* species.^[2]^

Thrombocytopenia is also frequently observed in malaria and has been highlighted as a significant clinical feature.^[3]^ While anemia is usually multifactorial – arising from hemolysis of parasitized and nonparasitized red blood cells (RBCs), dyserythropoiesis, and splenic sequestration – autoimmune hemolytic anemia (AIHA) is considered a rare complication.^[4,5]^

The pathogenesis of malaria-associated AIHA is believed to involve immune-mediated mechanisms, including complement activation and autoantibody production against erythrocyte surface antigens.^[6]^ A positive direct antiglobulin test (DAT) has been reported more frequently in acute malaria, reflecting the presence of malaria antigen–antibody complexes bound to RBCs.^[7]^ Thrombocytopenia in malaria is also thought to result from immune-mediated platelet destruction and splenic pooling.^[8]^

Although several case reports of *P vivax*-associated AIHA have been published, most describe the use of corticosteroids in addition to antimalarial therapy.^[9–16]^ Reports of successful resolution without corticosteroid therapy remain exceedingly rare. Here, we present a case of *P vivax* malaria complicated by severe AIHA and thrombocytopenia that resolved with antimalarial therapy, without immunosuppressive therapy.

## 2. Case presentation

A 28-year-old Ethiopian man presented to our hospital with a several-day history of fever, fatigue, and generalized weakness. On admission, his vital signs were: blood pressure 104/59 mm Hg, pulse rate 126/min, respiratory rate 22/min, and temperature 38.5°C. He appeared pale but was alert, conscious, and oriented.

**Figure 1.**
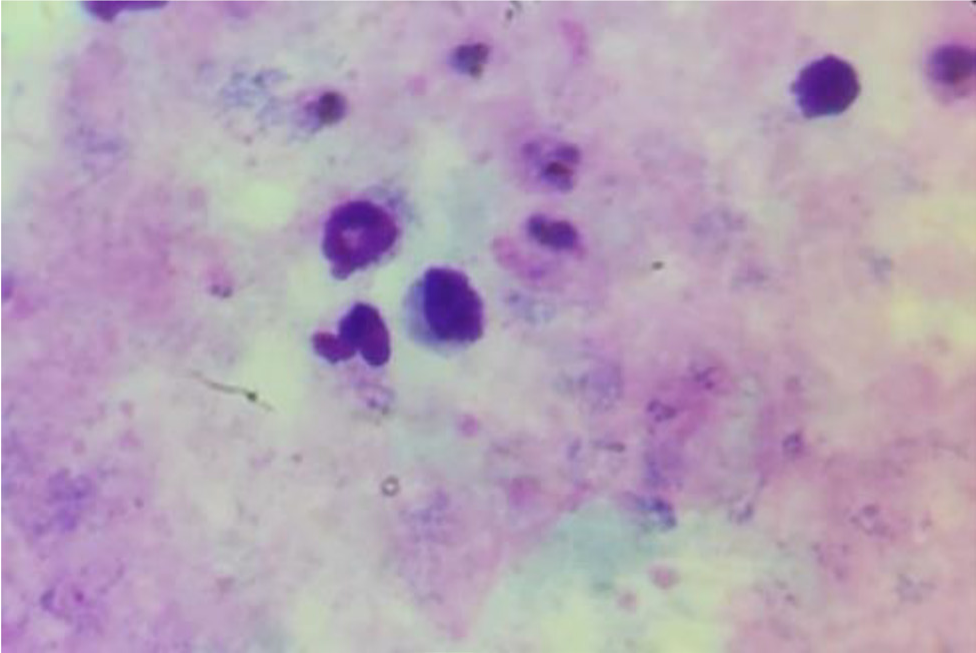
Peripheral blood smear showing ring forms and gametocytes of *Plasmodium vivax*.

Initial laboratory investigations showed severe anemia with a hemoglobin level of 6 g/dL (normocytic, normochromic), a leukocyte count of 7100/µL (68.6% neutrophils, 26.8% lymphocytes, and 3.6% monocytes), and thrombocytopenia with a platelet count of 88,000/µL. Total serum bilirubin was elevated at 194.2 µmol/L (direct bilirubin 151.3 µmol/L). A rapid malaria screening test was positive, and peripheral blood smears revealed ring forms and gametocytes of *P vivax* with a parasitemia index of 2% (Fig. 1).

**Figure 2.**
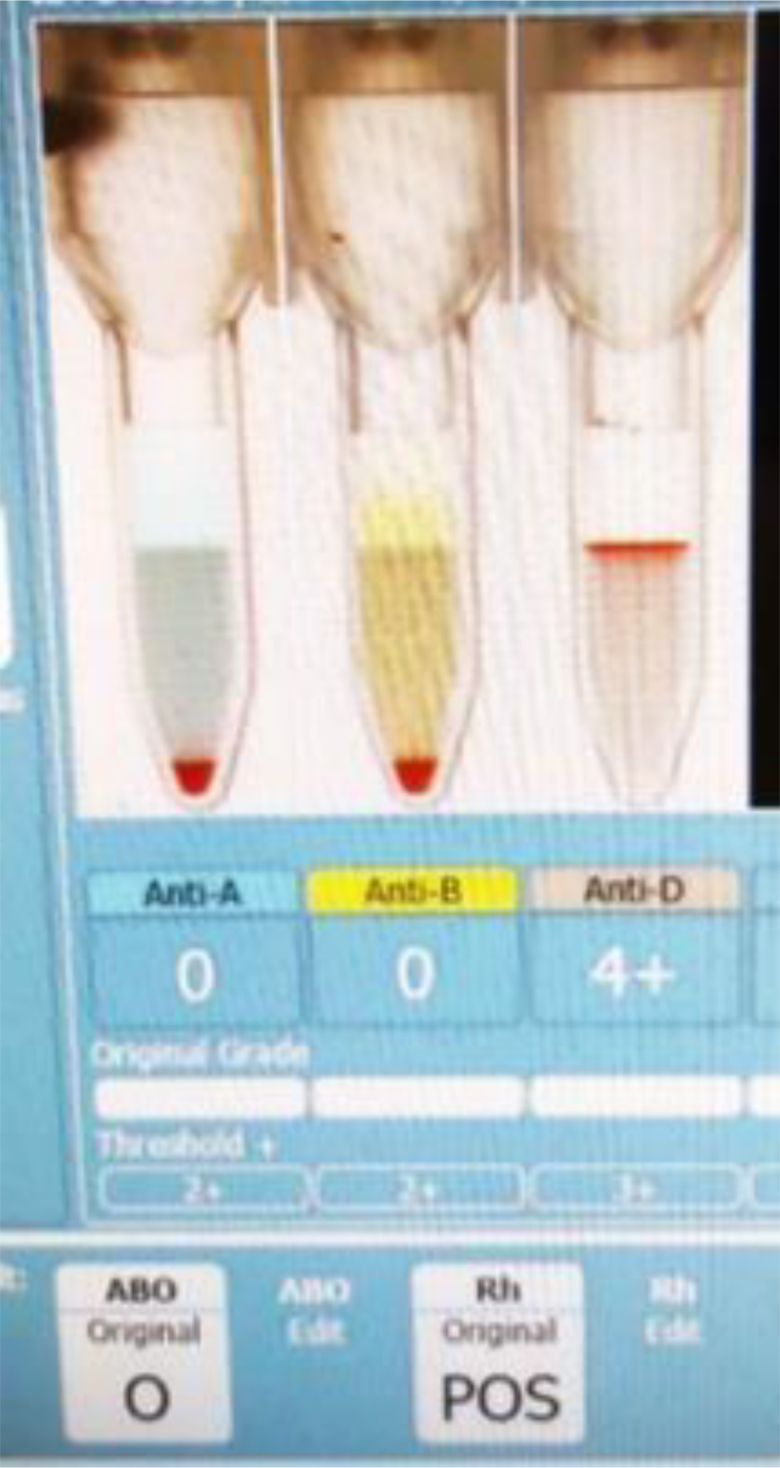
Blood grouping result showing O+ phenotype.

**Figure 3.**
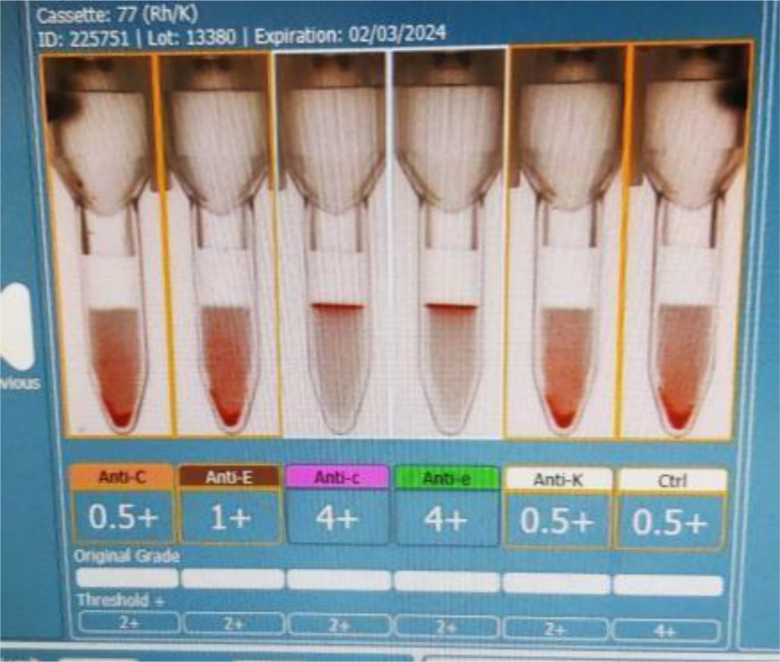
Extended cross-match panel demonstrating initial incompatibility.

Blood bank evaluation was requested for 2 units of packed RBCs. The patient’s blood group was determined as O+ (Fig. 2). Extended RBC antigen typing confirmed a cc ee K− phenotype (Figs. 8 and 9). Initial cross-matching with available O+ and O− cc ee K− units was incompatible (Fig. 3).

**Figure 4.**
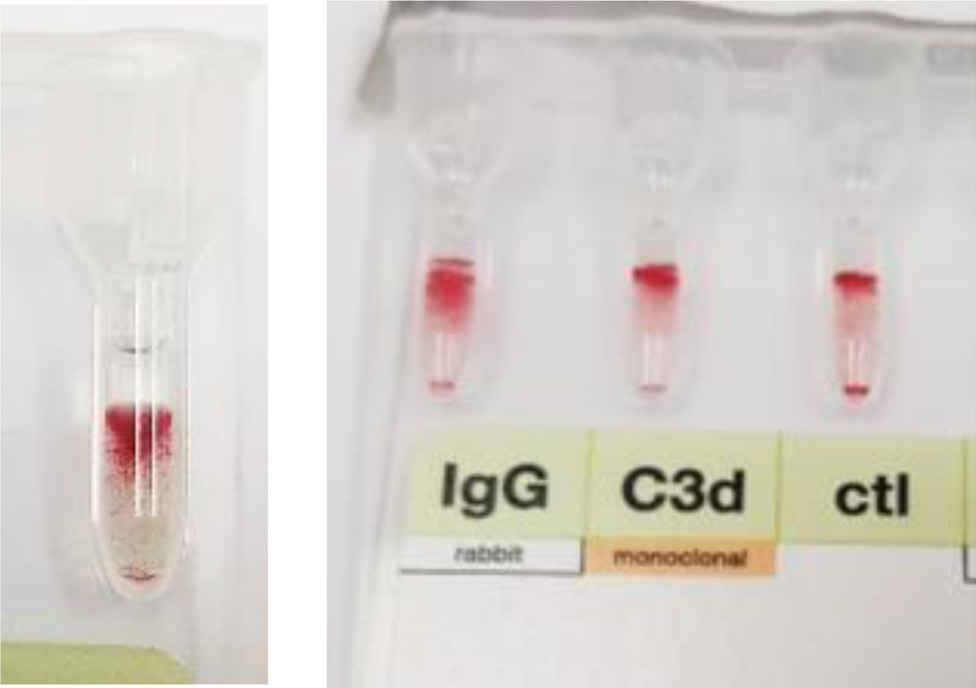
DAT showing strong positivity for both IgG and C3d at admission. C3d = complement component, DAT = direct antiglobulin test, IgG = immunoglobulin G.

**Figure 5.**
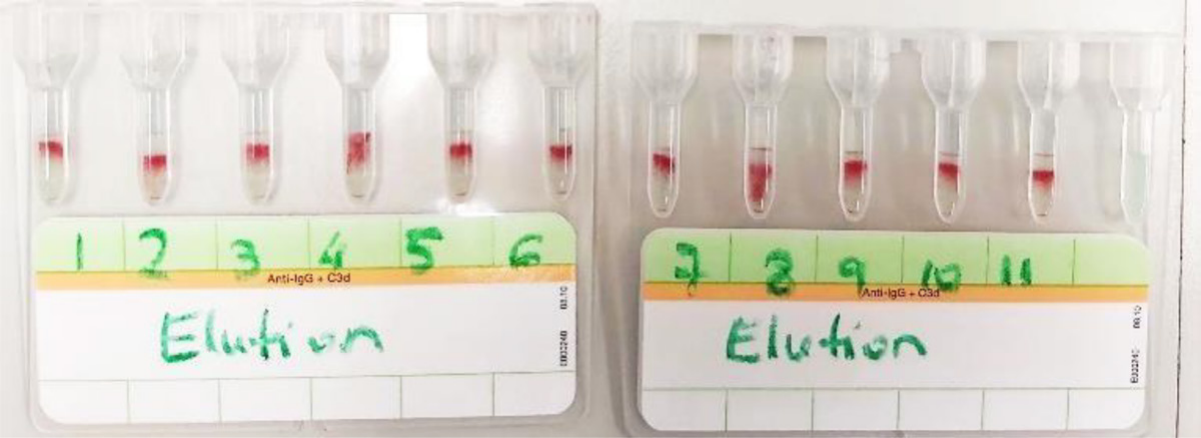
Elution test confirming the presence of antibodies bound to the patient’s red blood cells.

**Figure 6.**
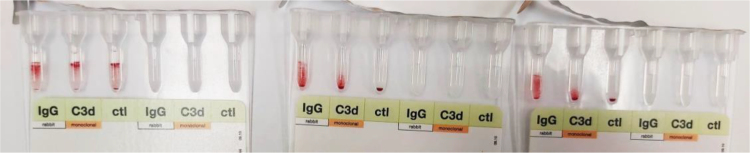
Serial DAT showing reduced reactivity after treatment compared to admission. C3d = complement component, DAT = direct antiglobulin test, IgG = immunoglobulin G.

**Figure 7.**
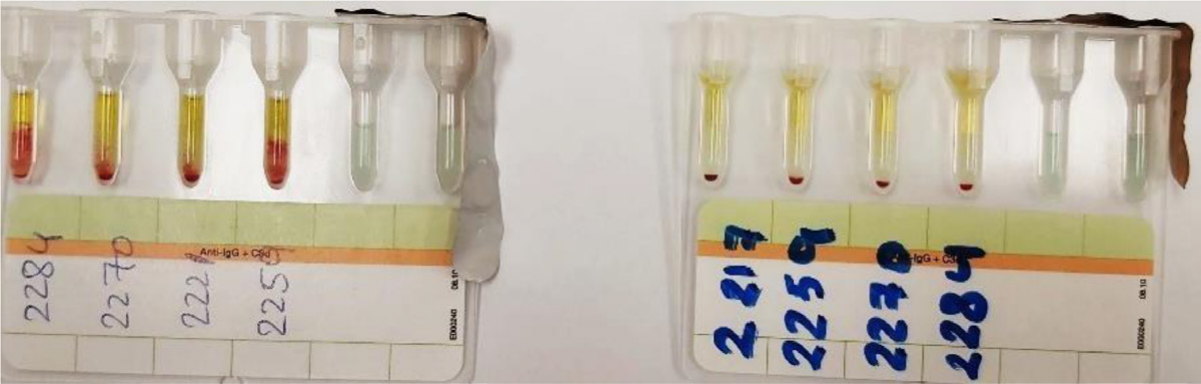
Adsorption test demonstrating removal of autoantibodies, enabling compatible cross-matching after repeated adsorptions.

**Figure 8.**
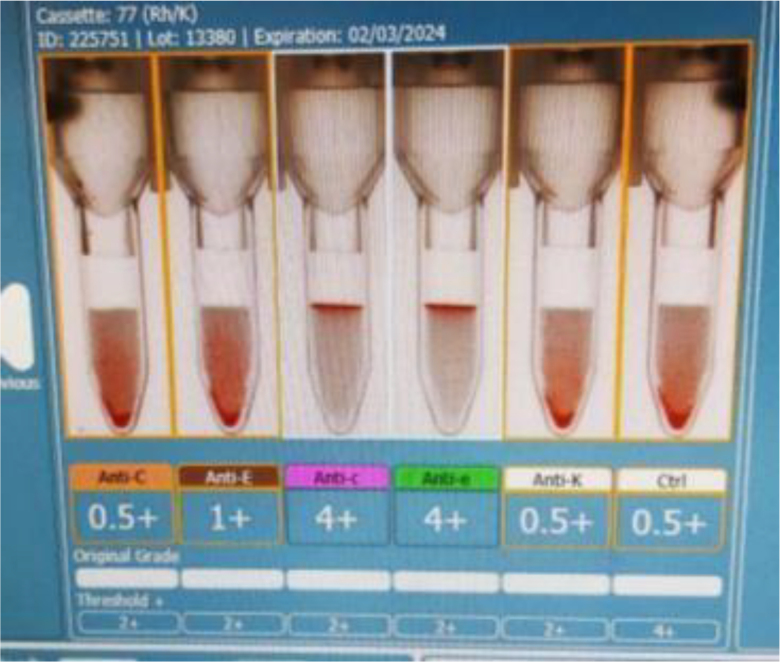
Extended red blood cell antigen typing panel confirming cc ee K− phenotype (panel 1).

**Figure 9.**
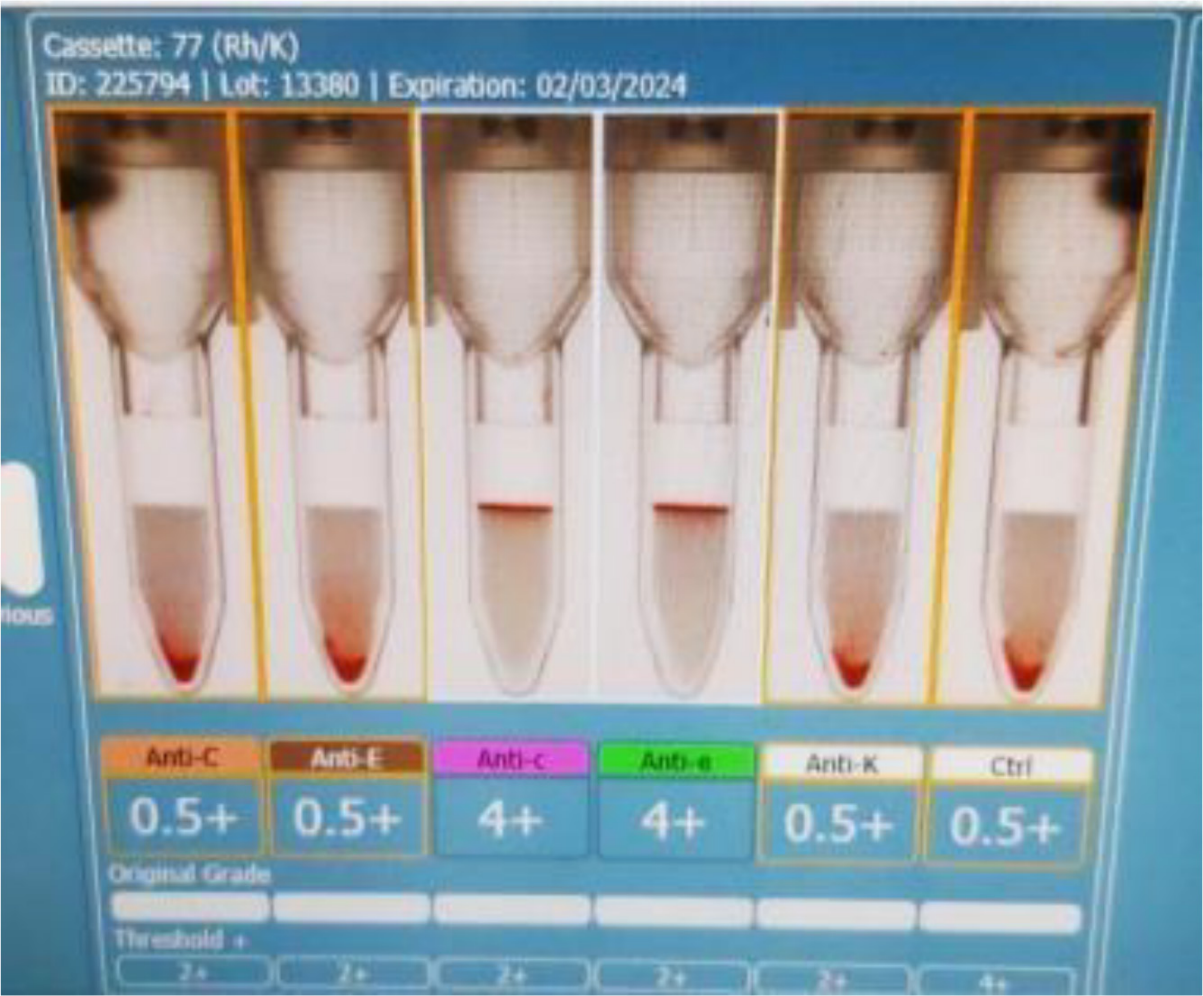
Extended red blood cell antigen typing panel confirming cc ee K− phenotype (panel 2).

The DAT was strongly positive for both immunoglobulin G (IgG) and complement component (C3d; Fig. 4). Elution testing was also positive, confirming the presence of antibodies bound to the patient’s RBCs (Fig. 5). Based on the positive immunohematological findings, including IgG and C3d positivity on the DAT, the findings were most consistent with a warm/mixed AIHA pattern. Adsorption techniques were subsequently performed, and after 3 rounds of allo-adsorptions, cross-matching became compatible (Fig. 7).

The patient received 3 compatible units of packed RBCs. He was treated with intravenous artesunate, followed by oral primaquine. No corticosteroids or platelet transfusions were administered.

By day 5 of treatment, hemoglobin had improved to 10.2 g/dL and platelet count normalized to 294,000/µL. Repeat DAT remained positive but with reduced intensity compared to admission (Fig. 6). Repeat cross-matching was compatible without further adsorption. Follow-up blood smears on day 5 showed gametocytes and a few ring forms, with a parasitemia index <1%. Rapid malaria antigen test results were negative.

The patient’s symptoms resolved, and he was discharged on day 6 in stable condition with a prescription for oral primaquine. Key laboratory findings during hospitalization are summarized in Table 1.

**Table 1:** Key laboratory findings during hospitalization.

Parameter	On admission	Day 5 of treatment	Reference range
Hemoglobin (g/dL)	6.0	10.2	13.5–17.5
WBC (×10^3^/µL)	7.1	–	4.0–11.0
Neutrophils (%)	68.6	–	40–75
Lymphocytes (%)	26.8	–	20–45
Monocytes (%)	3.6	–	2–10
Platelets (×10^3^/µL)	88	294	150–400
Total bilirubin (µmol/L)	194.2	–	<21
Direct bilirubin (µmol/L)	151.3	–	<5
DAT	3+	2+	Negative
Parasitemia index (%)	2.0	<1.0	–

The symbol “–” denotes not tested.

DAT = direct antiglobulin test, WBC = white blood cell.

## 3. Discussion

Malaria is well known to cause hematological complications such as anemia and thrombocytopenia.^[1–4]^ AIHA, however, remains an uncommon manifestation of malaria, particularly in *P vivax* infection.^[5]^ The mechanism of AIHA in malaria is not completely understood but is believed to involve immune-mediated processes such as malaria antigen–antibody complexes attaching to RBCs, complement activation, and autoantibody generation against erythrocyte surface proteins.^[6,7]^

Thrombocytopenia is also a common finding in malaria, reported in both *P vivax* and *P falciparum*, although more recent data suggest it may be more frequent in *P vivax* infections.^[3,8]^ The mechanism is multifactorial, but immune-mediated platelet clearance and splenic sequestration are likely contributors. In our patient, thrombocytopenia resolved rapidly following antimalarial therapy without platelet transfusion, consistent with a reversible immune-mediated mechanism.

Our case is notable for the comprehensive immunohematological evaluation that confirmed malaria-associated AIHA. The DAT was strongly positive for both IgG and C3d (Fig. 4), elution studies demonstrated antibody-coated RBCs (Fig. 5), and serial DAT testing showed reduced intensity following treatment (Fig. 6). Adsorption studies were also required to achieve compatible cross-matching (Fig. 7). Furthermore, extended antigen typing confirmed the patient’s cc ee K− phenotype (Figs. 8 and 9), excluding alloantibody-related incompatibility. Together, these investigations provided robust evidence for immune-mediated hemolysis triggered by *P vivax* malaria.

Several case reports of *P vivax*-associated AIHA have been reported worldwide (Table 2). In most of these reports, corticosteroid therapy was given in addition to antimalarial treatment.^[9–16]^ This clinical presentation is unusual in that both AIHA and thrombocytopenia resolved following antimalarial therapy and supportive RBC transfusion alone, without immunosuppressive therapy, including corticosteroids. This suggests that in selected patients, corticosteroid therapy may not always be required.

**Table 2:** Previously reported cases of *Plasmodium vivax*-associated autoimmune hemolytic anemia.

Author (yr)	Country	Age/sex	Treatment	Outcome
Lee (2008)	Korea	20/M	Antimalarial drugs	Improved
Sitcharungsi (2011)	Thailand	7 mo/M	Antimalarial + corticosteroids	Improved
Singh (2012)	India	35/F	Antimalarial + corticosteroids	Improved
Sharma (2012)	India	15/F + 7/F	Antimalarial + corticosteroids	Improved
Sonani (2013)	India	20/M	Antimalarial + corticosteroids	Improved
Ghosh (2017)	India	12 d/M	Antimalarial + corticosteroids	Improved
Taneja (2019)	India	6 mo/M	Antimalarial + corticosteroids	Improved
Nishith (2022)	India	15/M	Antimalarial + corticosteroids	Improved
Present case (2024)	Saudi Arabia	28/M	Antimalarial only (no corticosteroids)	Recovered

Animal studies with *Plasmodium* and *Babesia* species have documented the presence of anti-erythrocyte and antiplatelet antibodies, strengthening the hypothesis of immune-mediated cytopenias in parasitic infections.^[17]^ In addition, a recent murine study confirmed the development of anti-erythrocyte and antiplatelet autoantibodies following *Plasmodium* infection, further supporting immune-mediated mechanisms in malaria-associated hematological complications.^[17]^ An older report from Myanmar also described AIHA complicating both *P falciparum* and *P vivax* infections.^[18]^

## 4. Conclusion

This case highlights a rare presentation of *P vivax* malaria complicated by severe AIHA and thrombocytopenia. Unlike most previous reports, our patient achieved complete recovery with antimalarial therapy and supportive transfusion alone, without immunosuppressive therapy. Importantly, the diagnosis was confirmed through a comprehensive immunohematological evaluation, including DAT, elution, adsorption, and antigen typing, providing robust evidence of immune-mediated hemolysis. This observation underscores the importance of early recognition of hematological complications in malaria and suggests that, in selected patients, corticosteroids may not always be required for resolution.

## Acknowledgments

The authors extend their appreciation to Umm Al-Qura University, Saudi Arabia for funding this research work through grant number: 26UQU4420118GSSR02.

## Author contributions

**Conceptualization:** Abdu Aldarhami.

**Data curation:** Abdu Aldarhami, Rahmah Alzaylaee, Ibrahim Awadh, Raja Sassi, Hassan Alzahrani, Ali Alsamiri, Kamal Ismail, Nizar H. Saeedi, Mohammed M. Jalal, Abdullah Yahya Alrashdi, Abdulrahman S. Bazaid.

**Funding acquisition:** Abdu Aldarhami.

**Investigation:** Abdu Aldarhami, Rahmah Alzaylaee, Ibrahim Awadh, Raja Sassi, Hassan Alzahrani, Ali Alsamiri, Kamal Ismail, Nizar H. Saeedi, Mohammed M. Jalal, Abdullah Yahya Alrashdi, Abdulrahman S. Bazaid.

**Project administration:** Abdu Aldarhami, Ibrahim Awadh, Abdullah Yahya Alrashdi.

**Supervision:** Abdu Aldarhami, Ibrahim Awadh, Abdullah Yahya Alrashdi.

**Writing – original draft:** Abdu Aldarhami, Rahmah Alzaylaee.

**Writing – review & editing:** Abdu Aldarhami, Rahmah Alzaylaee, Ibrahim Awadh, Raja Sassi, Hassan Alzahrani, Ali Alsamiri, Kamal Ismail, Nizar H. Saeedi, Mohammed M. Jalal, Abdullah Yahya Alrashdi, Abdulrahman S. Bazaid.

**Formal analysis:** Rahmah Alzaylaee.

**Visualization:** Abdulrahman S. Bazaid.
